# Forward-Focused Coping Predicts Better Mental Health Outcomes in Older Adults During COVID-19 Pandemic

**DOI:** 10.1093/geroni/igaa057.3465

**Published:** 2020-12-16

**Authors:** Leslie Jordan, John Woodard, Gabriel Pena, Naomi Arnold-Nedimala, Junyeon Won, Daniel Callow, J Carson Smith

**Affiliations:** 1 University of Maryland, College Park, College Park, Maryland, United States; 2 Wayne State University, Detroit, Michigan, United States

## Abstract

Psychosocial stressors associated with the COVID-19 pandemic may increase risk of depression and anxiety in the general population. Older adults may be especially vulnerable to these psychosocial stressors and their impact on mental health outcomes. Consequently, there is an urgent need to identify protective factors for older adults. The purpose of the present study is to determine the relative contribution of coping flexibility and two distinct coping strategies, forward-focused and trauma-focused, on negative affect in older adults during the COVID-19 pandemic. Data were collected using an online survey, including questions about demographic information, coping, depression, and anxiety. Participants aged 50 and over were included in our analyses of depression (N = 800) and anxiety (N = 638). Results indicate that both higher coping flexibility and higher forward-focused coping predict lower depression and lower anxiety. In contrast, higher trauma-focused coping predicts slightly higher depressive symptoms but is not a significant predictor of anxiety. Our findings suggest that higher forward-focused coping may serve as a protective factor in older adults during the pandemic and, therefore, may be an effective treatment target for mental health interventions.

